# Expression of Tailored α-N-Acetylglucosaminidase in *Escherichia coli* for Synthesizing Mannose-6-Phosphate on N-Linked Oligosaccharides of Lysosomal Enzymes

**DOI:** 10.3390/bioengineering12040425

**Published:** 2025-04-17

**Authors:** Yunsong Cao, Wei Wang

**Affiliations:** 1 State Key Laboratory of Bioactive Substance and Function of Natural Medicines, Institute of Materia Medica, Chinese Academy of Medical Sciences & Peking Union Medical College, Beijing 100050, China; caoyunsong2016@163.com; 2 Key Laboratory of Biosynthesis of Natural Products of National Health Commission of the Peoples Republic of China, Institute of Materia Medica, Chinese Academy of Medical Sciences & Peking Union Medical College, Beijing 100050, China

**Keywords:** lysosomal enzyme, uncovering enzyme, mannose-6-phosphate, maltose-binding protein (MBP)

## Abstract

Lysosomal enzymes are synthesized as N-glycosylated glycoproteins with mannose-6-phosphate (M6P) moieties, which are responsible for their binding to M6P receptors and transporting to the lysosome. In the M6P biosynthetic pathway, a Man_8_GlcNAc_2_ glycoform is converted to M6P groups through two consecutive enzymatic reactions, including N-acetylglucosamine (GlcNAc)-1-phosphotransferase (GNPT), transferring GlcNAc-1-phosphate from UDP-GlcNAc to the C6 hydroxyl groups of mannose residues, and then, removal of the covering GlcNAc moiety from the GlcNAc-P-mannose phosphodiester was carried out using an α-N-acetylglucosaminidase (referred to as ‘uncovering enzyme’, UCE) in the *trans*-Golgi network (TGN). Here, we expressed differently tailored versions of the UCE, including four truncated variants, in *Escherichia coli*. The four variants with the signal peptide, transmembrane domain, propiece and cytoplasmic tail truncated, respectively, were purified by affinity chromatography, and their enzymatic activities were assayed using a UDP-Glo kit. By fusing a maltose-binding protein (MBP) in the N-terminus of the UCE variants, the fusion proteins could be soluble when expressed in *E. coli*. The highest concentration of the purified enzyme was 80.5 mg/L of fermentation broth. Furthermore, the UCE with the core catalytic domain exhibited the highest uncovering activity.

## 1. Introduction

**Figure 1:**
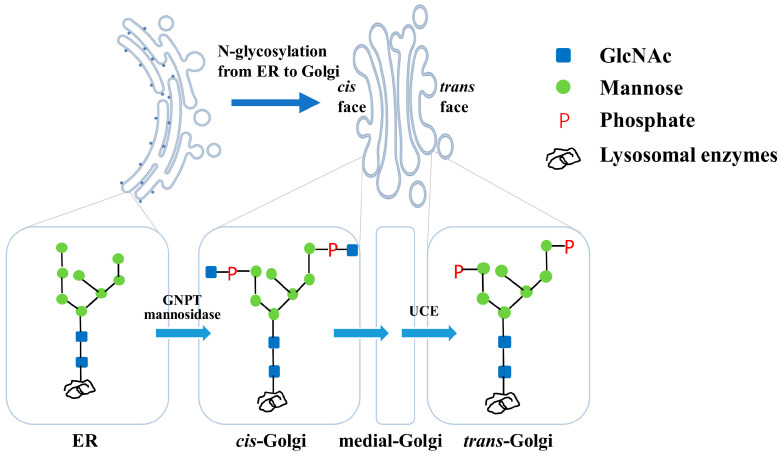
Biosynthetic pathway of mannose-6-phosphate in lysosomal enzymes. An N-glycan with Man_8_GlcNAc_2_ was first synthesized on lysosomal enzymes in the ER. Next, the two GlcNAc-phosphate groups were conjugated by GNPT in *cis*-Golgi. Finally, the two terminal GlcNAcs were uncapped by the UCE in *trans*-Golgi to form the M6P glycoform.

Lysosomal enzymes, an array of acidic hydrolases, are involved in degrading metabolites, including proteins, polysaccharides and lipids [1,2,3]. All lysosomal enzymes are glycoprotein and are glycosylated in the endoplasmic reticulum (ER) and Golgi apparatus. The formation of a mannose-6-phosphate (M6P) glycoform on the N-glycosylation sites is the prerequisite for the enzymes being recognized by the cation-dependent M6P receptors on *trans*-Golgi and transported to the lysosome [4,5]. The M6P synthesis involves three consecutive catalytic reactions. Firstly, the enzymes are glycosylated in the ER to form a glycoform with two N-acetylglucosamines (GlcNAc) and eight mannoses (referred to as Man_8_GlcNAc_2_) and then transported into the *cis*-Golgi compartment. Next, two GlcNAc-1-phosphate groups are added onto the termini of two α-1,2-mannose residues by GlcNAc-1-phosphotransferase (GNPT) in *cis*-Golgi. Finally, the two masking GlcNAcs are uncapped by N-acetylglucosamine-1-phosphodiester α-N-acetylglucosaminidase, also known as the uncovering enzyme (UCE), in the *trans*-Golgi network (TGN) [4,5] (Figure 1). Therefore, the M6P tags are exposed, and the enzymes can be recognized by M6P receptors.

**Figure 2:**
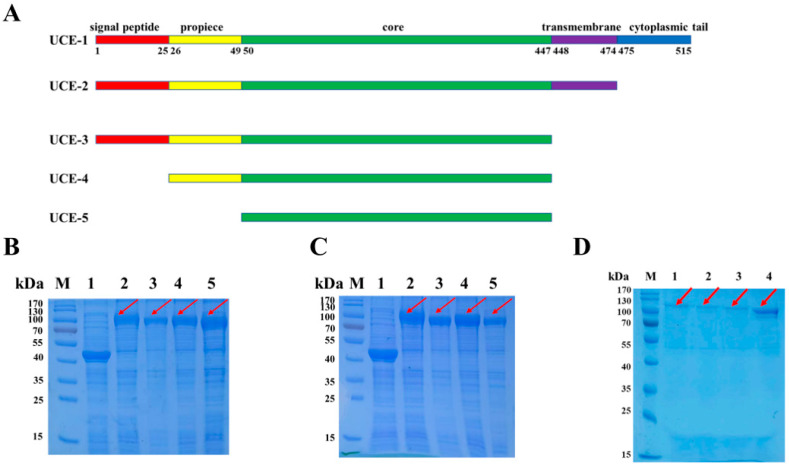
The design, expression and purification of the UCE variants. (**A**) Schematic of the structures of the UCE variants. UCE-2 is a variant without a cytoplasmic tail. UCE-3 is a variant without a transmembrane domain and cytoplasmic tail. UCE-4 is a variant without a signal peptide, transmembrane domain and cytoplasmic tail. UCE-5 is a minimal variant with only the core remained. The ratio of each domain was not drawn to scale. (**B**) SDS-PAGE analysis of the expression of UCE variants in the supernatant. (**C**) SDS-PAGE analysis of the expression of UCE variants in the cell pellet. Lane 1, pCold-MBP empty vector. Lanes 2–5: UCE-2 to UCE-4. (**D**) SDS-PAGE analysis of the recombinant UCE, eluted with a buffer containing 300 mmol/L imidazole. Lanes 1–4: UCE-2 to UCE-5. The red arrows represent the band of MBP-UCE.

As one of the two key enzymes for M6P synthesis in the Golgi apparatus, human UCE is a typical type I transmembrane protein with 515 amino acid residues, encoded by the *NAGPA* gene on chromosome XⅥ [6,7]. It has a 24-amino acid signal peptide on the N-terminus, followed by a 24-amino acid propiece, a core region (from 50 to 447 amino acid residues of primary sequence), and a C-terminal transmembrane region (positions 448 to 474). In addition, amino acid residues of 475 to 515 form a cytoplasmic tail in the C-terminus (Figure 2A). The UCE is initially synthesized as an inactivated form. After being cleaved between Arg49 and Asp50, the propiece is removed to form a mature UCE. The cleavage takes place in the TGN by Furin, a protease which recognizes a consensus sequence of RARLPR↓D [6,7,8,9].

As a transmembrane protein, although UCE has been functionally expressed and characterized in mammalian COS, CHO cells and insect cells [7,9] and also used to investigate the in vivo M6P-elaboration processing of lysosomal α-L-iduronidase (IDUA) in plant cells [10], its costly production and low amounts limit its widespread use for in vitro M6P glycoform synthesis. In addition to this, as an expression construct, the complex maturation mechanism makes it difficult to construct a heterologously biosynthetic expression system (e.g., engineered yeast). *Escherichia coli* is the most frequently applied prokaryotic expression system, with its advantages including ease of cultivation, a rapid growth rate, a low cost and high productivity compared with mammalian, insect and plant cells [11]. Here, we reported the soluble expression of four truncated forms of UCEs, fused with a maltose-binding protein (MBP) tag [12,13] in *E. coli*, and determined the GlcNAc uncovering activity of the enzymes. The results showed that only the UCE without a propiece can achieve a high uncovering activity. By the expression and purification of UCEs, a methodology of in vitro glycosylation modification will be established for producing lysosomal enzymes with M6P.

## 2. Materials and Methods

### 2.1. Materials

*E. coli* strain DH10b-competent cells were prepared and stored by our lab. The host strain SHuffle T7 was purchased from NEB (Ipswich, MA, USA). The expression vector pCold-MBP was purchased from Zoman Biotechnology (Beijing, China). The UDP-Glo kit for measuring the UCE activity was obtained from Promega (Madison, WI, USA). UDP-GlcNAc, the substrate of UCE, was obtained from Sigma (Virginia Beach, VA, USA).

### 2.2. Design of the UCE Variants

To determine the effects of each domain of UCE on its activity, four truncated variants, referred to as UCE-2, UCE-3, UCE-4 and UCE-5, were designed as follows (Figure 2A): UCE-2 is a 51.4 kDa variant without the cytoplasmic tail. The variant UCE-3 (48.6 kDa) is truncated by deletion of the transmembrane domain and cytoplasmic tail. Compared with UCE-3, UCE-4 (45.9 kDa) is further simplified by not having a signal peptide. UCE-5 is a minimal variant that only has the core region, the size of which is 43.2 kDa.

### 2.3. Cloning of the UCE Variants and Construction of the Expression Vectors

A full-length human UCE gene was synthesized by Sangon Biotech (Shanghai, China) and cloned into the plasmid pGH as a template. The encoding regions of UCE-2, UCE-3, UCE-4 and UCE-5 were amplified using the primer pairs of UCE-MBP_BamH1-1/UCE_Sal1-2, UCE-MBP_BamH1-1/UCE_Sal1, UCE-MBP_BamH1-4/UCE_Sal1 and UCE-MBP_BamH1/UCE_Sal1, respectively. Then, the amplified DNA fragments of four UCE variants were inserted into the BamH I and Sal I sites of pCold-MBP using the ClonExpress^®^ II One Step Cloning Kit (Vazyme, Nanjing, China). The ORFs of four MBP-UCE expression vectors, pCold-MBP-UCE-2/UCE-3/UCE-4/UCE-5, were verified via colony PCR and sequencing (Sangon Biotech, Shanghai, China). The primers used in this study are listed in Table 1.

### 2.4. The Expression of UCE Variants

A total of 100 ng of four expression vectors was transformed into SHuffle T7-competent cells. The cells were spread onto an LB–ampicillin (100 μg/mL) plate and incubated overnight. A single colony was selected, inoculated into 5 mL LB broth with ampicillin and cultured overnight at 37 °C with agitation at 220 rpm. Then, 1 mL of the overnight culture was transferred to 100 mL of fresh LB broth with ampicillin and cultured at 37 °C and 220 rpm until the optical density at 600 nm (OD600) reached 0.4 to 0.6. The culture broth was incubated at 15 °C for 30 min. Then, 0.5 mmol/L of isopropyl-β-D-thiogalactoside (IPTG) was added to induce the expression of the target protein. After overnight culture at 15 °C and 150 rpm, 100 mL of culture was centrifuged, and the cell pellets were resuspended in 10 mL of lysis buffer (25 mmol/L Tris-HCl and 150 mmol/L NaCl; pH 7.5) and then sonicated on ice. The lysed cells were centrifuged at 4 °C, and the protein expressions in the supernatant and cell pellets were analyzed by sodium dodecyl sulfate–polyacrylamide gel electrophoresis (SDS-PAGE).

### 2.5. The Purification of Recombinant UCE Proteins

The fusion protein could be purified by Ni^2+^ affinity chromatography, because a His-tag was fused to the N-terminus of MBP. The supernatant of the lysed cells was loaded onto a column packed with Ni-NTA resin (CoWin Biosciences, Shanghai, China) and incubated at 4 °C. The resin was washed with buffers containing 0, 20, 50, 100 and 200 mmol/L imidazole, respectively, in order to remove the impurity proteins. Then, the resin was eluted with a buffer containing 300 mmol/L imidazole to collect the pure MBP-UCE variants. The eluents were used for further activity tests. The recovery yield of the UCE fusion proteins was measured by a NanoReady spectrophotometer (Life Real, Hangzhou, China) at 280 nm (A280).

### 2.6. Determination of the Uncovering Activity of MBP-UCE Variants

A total of 1.5 μg of the purified protein was mixed in a 96-well plate with a buffer (25 mmol/L Tris-HCl and 150 mmol/L NaCl; pH 7.5) and UDP-GlcNAc to a total volume of 25 μL. The final concentration of UDP-GlcNAc was 100 μmol/L. The plate was incubated at 30 °C with agitation at 200 rpm for 30 min. According to the instructions for the UDP-Glo kit, 25 μL of UDP detection reagent was added immediately, and the plate was incubated at 30 °C and 200 rpm for another 1 h. The luminescence value was read in a microplate reader.

### 2.7. Statistical Analysis

The calibration curve and activity assay experiment were performed with triplicate samples. The data in the activity assay were presented as mean ± standard deviation (SD). The visualization of the data was conducted by GraphPad Prism 8.0 (GraphPad Software, San Diego, CA, USA).

## 3. Results

### 3.1. The Expression of UCE Variants in E. coli

Four truncated variants, referred to as UCE-2, UCE-3, UCE-4 and UCE-5, were constructed (Figure 2A), and the corresponding DNA fragments were sub-cloned into pCold-MBP. Given that a UCE harbors a transmembrane domain at its C-terminus [6,7,8,9], some variants might be insoluble when expressed in *E. coli*. Thus, a widely applied soluble tag, MBP [12,13], was fused to the N-terminus of UCE to produce soluble proteins. After overnight incubation, the proteins of the supernatant and the cell pellets were analyzed by SDS-PAGE and Coomassie Brilliant Blue staining, respectively (Figure 2B,C). A band of ~100 kDa (MBP tag plus UCE variants, lanes 2 to 5 in Figure 2B,C) was detected in both the lytic supernatant and cell pellets of all the four variants. This proves that the four UCE variants, even with a transmembrane domain, can be soluble when fused with an MBP tag.

### 3.2. The Purification of UCE Variants via Ni^2+^-Based Immobilized Metal Ion Affinity Chromatography

The vector pCold-MBP harbors a His-tag on the N-terminal of the MBP for affinity purification of the MBP-fused protein. The supernatants of UCE-2 to UCE-5 were loaded onto Ni-NTA resin, followed by elution with buffers containing low concentrations of imidazole to remove the unwanted proteins. Then, the resin was eluted with a buffer containing 300 mmol/L imidazole, and the eluent containing pure UCE variants was pooled. SDS-PAGE was performed for detection of UCE-2 to UCE-5 in the eluates (Figure 2D). A major band of ~100 kDa was detected in all four variants, which is consistent with the expected sizes of the corresponding MBP-UCE variants. These results showed that UCE-2 to UCE-5 could easily be purified as a fusion protein by affinity chromatography in this design. The concentrations of the purified UCE-2, UCE-3, UCE-4 and UCE-5 were about 36.9, 21.7, 19.3 and 80.5 mg/L fermentation broth, respectively.

### 3.3. The Uncovering Activity Assay of UCE Variants

**Figure 3:**
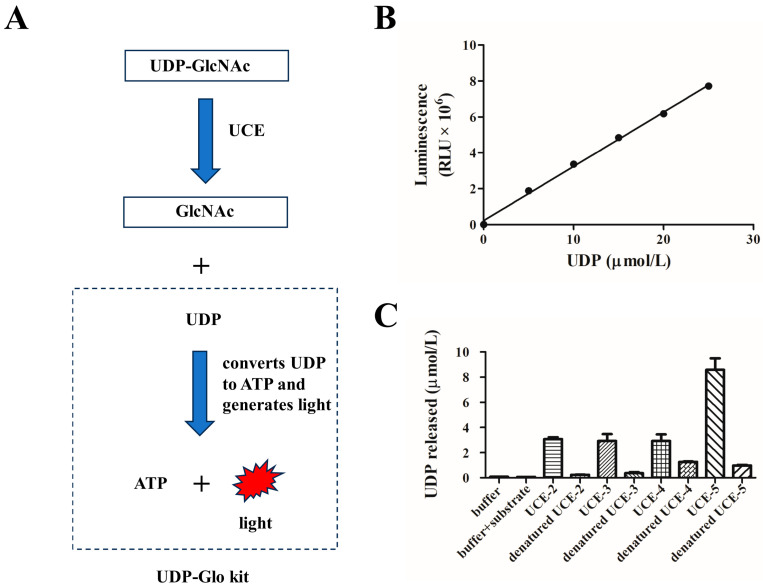
Determination of the activities of UCE variants. (**A**) A schematic of a method for detecting the titer of UDP released by a one-step UCE hydrolysis reaction using the UDP-Glo kit. The UDP detection reagent in the kit can convert UDP into ATP and generate light. The luminescence can reflect the titer of the UDP. (**B**) Luminescence–UDP concentration calibration curve. (**C**) UDP released in reactions using the UCE variants, where denatured enzymes were calculated according to the calibration curve. Reactions with buffer only, and with buffer and substrate (without enzyme), were set as blank and negative control, respectively. The UCE activity is shown as UDP released per micromole of the enzyme.

As mentioned above, the UCE has the ability to hydrolyze the masking GlcNAc group on the mannose phosphate moiety [5,8]. It is difficult to evaluate the uncovering activity directly by using glycans on lysosomal enzymes as the substrate. Alternatively, we chose uridine diphosphate–GlcNAc (UDP-GlcNAc) as the substrate. UDP-GlcNAc, a sugar nucleotide, is a common glycosyl donor in vivo and in vitro for GlcNAc transferase [14]. UDP-GlcNAc can be hydrolyzed and converted into UDP and GlcNAc by UCE. The UDP-Glo kit was used to convert UDP to ATP through a one-step reaction and generate light [10] (Figure 3A). The luminescence intensity was detected and reflected the titer of the released UDP.

Different concentrations of standard UDP were prepared and added to the UDP detection reagent. The luminescence was read, and a calibration curve was established (y = 306205x + 216027, R^2^ = 0.9974, Figure 3B). Then, the four purified MBP-UCE variants were incubated with 100 μmol/L UDP-GlcNAc, followed by adding a UDP detection reagent. As is shown in Figure 3C, the reaction with UCE-5 released the maximum amount of UDP among the four variants, which revealed that UCE-5 exhibited the highest activity. However, the other three variants with the propiece still exhibited moderate uncovering activities. Therefore, the cleavage of the propiece is of vital importance for the uncovering activity of a UCE. It is worth noting that the fusion proteins without removal of the N-terminal MBP still demonstrated high uncovering activities in this study. Therefore, this might provide another new approach for expressing other solubility-enhancing proteins.

## 4. Discussion

It has been reported that UCE comprises the transmembrane domain at its C-terminus, which makes it difficult to express the full-length protein. Therefore, the functional expression and characterization of UCE in mammalian cells [7], insect cells [9] and plant cells [10] were all conducted using the C-terminal truncated form. Among these reports, Zeng et al. [10] heterologously expressed UCE with the propiece intact in *Arabidopsis thaliana* and demonstrated that mature UCE was produced. This means that a Furin-like protease exists in plants. However, in this study, an intact MBP-UCE fusion protein (~100 kDa), instead of the propiece-removed species, was detected (Figure 2B,C), indicating that no Furin-like protease might exist in *E. coli*. Furthermore, an MBP tag improved the solubility of the UCE, as well as preserving its activity when fused in its N-terminus. Hence, it provided a useful methodology for expressing active proteins with transmembrane domains and without the removal of the MBP tag.

Recently, Gorelik et al. [8] determined the crystal structure of the propiece-autoinhibited catalytic domain of guinea pigs, which has 90% similarity to human UCE. This revealed that the propiece binds in a groove on the UCE globular catalytic domain, blocking the active site access. In addition, UCE is able to hydrolyze UDP-GlcNAc, which is the substrate of GNPT. This would inhibit mannose–phosphate–GlcNAc synthesis if UCE is active in the *cis*-Golgi compartment. It can be figured out that this is the reason for UCE remaining inactive until reaching TGN and being cleaved by Furin [9]. Therefore, the removal of this inhibitory propiece enables the uncovering activity, which is in accordance with our results that the enzymatic activities of UCE2 to UCE4 are partially reduced by the existing propeptide, and the recombinant MBP, which is different from the inhibitory segment, has no effect on the catalytic activity of UCE-5, which has the highest relative activity (Figure 3C). As well as the inhibitory propiece existing in the UCE-2 to UCE-4 variants resulting in the biological activity discrepancy between UCE-2 to UCE-4 and UCE5, the structural instability factors cannot be ruled out, because there is a cysteine-rich C-terminal domain (CTD) in UCE, and the existence of the pro-peptide leads to the formation of incorrect disulfide bonds due to *E. coli* lacking the reducing capacity to form correct disulfide bonds.

The N-glycan on lysosomal enzymes is the natural substrate of UCE, making it difficult to evaluate UCE activity. Previously, the UCE activity was measured by using isotope-labeled [^3^H]GlcNAc-P-ManαMe as a substrate [15]. Here, we introduced a method for the measurement of UCE activity utilizing the UDP-Glo kit [10], avoiding the use of an isotope-labeled substrate (Figure 3A).

Lysosomal enzymes have successfully been expressed in mammalian cells for producing enzyme replacement therapy (ERT) drugs for treating lysosomal storage disorders (LSDs). Nevertheless, some enzymes are not phosphorylated well naturally in the host cells, leading to a low M6P content in the drugs [16,17]. For example, only 0.7 mol of M6P per mol enzyme was observed in Aglucosidase alfa (α-glucosidase for treating Pompe disease) [16]. To address the challenges of production costs and potential viral contamination associated with mammalian systems, plant-based platforms have emerged as innovative alternatives for biopharmaceutical production, including lysosomal enzymes [18,19]. Nevertheless, these platforms present a biochemical constraint: plant-synthesized lysosomal enzymes predominantly exhibit high-mannose-type N-glycans due to the absence of endogenous M6P-tagging enzymatic machinery. In a pioneering study, Zeng et al. [10] successfully established proof-of-concept co-expression of human M6P modification system components (GNPTαβ, GNPTγ and UCE) with the therapeutic target iduronidase (IDUA) in plant hosts. In addition, an in vitro phosphorylation method using a recombinant soluble GNPT and UCE has been explored [9,20,21]. In order to synthesize M6P in vitro, a sufficient amount of UCE is required to be produced. Although UCE has been successfully expressed in mammalian, insect and plant cells [7,8,9,10], its expression levels were not mentioned, indicating that a high-producing platform host is yet to be established. In this study, *E. coli* was selected to express UCE, and a considerable expression level of 80.5 mg/L was achieved. Therefore, the in vitro modification of lysosomal enzymes by GNPT and UCE will make it possible to increase the M6P content of lysosomal enzymes in order to increase the therapeutic efficacy. In this study, the yields of recombinant MBP-UCE with high uncovering activity can be further improved by optimizing the fermentation parameters. To the best of our knowledge, as a tool enzyme of in vitro M6P elaboration process, this research work represents a major step forward for constructing an alternative scheme to increase the M6P content of lysosomal enzymes in the future.

## 5. Conclusions

In conclusion, we successfully expressed and purified four truncated versions of UCE in *E. coli*, of which the propiece-removed form exhibited the highest activity. Through the platform established in this study, UCE can be produced in a large amount and easily purified in order to modify various lysosomal enzymes in vitro. This study will continue to develop an alternative for producing ERT drugs with higher efficiency in treating lysosomal storage disorders.

## Figures and Tables

**Table 1:** PCR primers used in this study.

Primers	Oligonucleotide Sequences (5′-3′)
UCE-MBP_BamH1	GTACCCTCGAGGGATCCGAGAATCTGTACTTCCAAGGAGGAGATTGCACACGTGTTCGT
UCE_Sal1	TACCTATCTAGACTGCAGGTCGACTCATGTACGAGTGAAAAAAGACAATT
UCE-MBP_BamH1-1	GTACCCTCGAGGGATCCGAGAATCTGTACTTCCAAGGAGGAATGGCTACTTCTACAGGT
UCE-MBP_BamH1-4	GTACCCTCGAGGGATCCGAGAATCTGTACTTCCAAGGAGGATTAGATTCTGGTGCTTCT
UCE_Sal1-2	TACCTATCTAGACTGCAGGTCGACTCAAGACAATAACAAAGACAAGTTTG

## Data Availability

Data are contained within the article.
